# Corona Isolation Method Matters: Capillary Electrophoresis Mass Spectrometry Based Comparison of Protein Corona Compositions Following On-Particle versus In-Solution or In-Gel Digestion

**DOI:** 10.3390/nano9060898

**Published:** 2019-06-20

**Authors:** Klaus Faserl, Andrew J. Chetwynd, Iseult Lynch, James A. Thorn, Herbert H. Lindner

**Affiliations:** 1 Division of Clinical Biochemistry, Medical University of Innsbruck, Innrain 80-82, A-6020 Innsbruck, Austria; Klaus.Faserl@i-med.ac.at; 2 AB Sciex UK Ltd., Phoenix House, Lakeside Drive, Warrington, Cheshire WA1 1RX, UK; a.j.chetwynd@bham.ac.uk (A.J.C.); Jim.Thorn@sciex.com (J.A.T.); 3 School of Geography Earth and Environmental Sciences, University of Birmingham, Edgbaston, Birmingham B15 2TT, UK; I.Lynch@bham.ac.uk

**Keywords:** CE-MS, mass-spectrometry, nanoparticles, proteomics, protein corona, reproducibility, capillary electrophoresis

## Abstract

Increased understanding of the role of the nanomaterial protein corona in driving nanomaterial uptake into, and impacts on, cells and organisms, and the consequent need for characterization of the corona, has led to a flourishing of methods for isolation and analysis of the constituent proteins over the past decade. However, despite over 700 corona studies to date, very little is understood in terms of which methods provide the most precise and comprehensive characterization of the corona. With the increasing importance of the modeling of corona formation and its correlation with biological impacts, it is timely to properly characterize and validate the isolation approaches used to determine the protein corona. The current work introduces Capillary Electrophoresis with Electro Spray Ionization Mass Spectrometry (CESI-MS) as a novel method for protein corona characterizations and develops an on-particle tryptic digestion method, comparing peptide solubilization solutions and characterizing the recovery of proteins from the nanomaterial surface. The CESI-MS was compared to the gold standard nano-LC-MS for corona analysis and maintained a high degree of reproducibility, while increasing throughput by >3-fold. The on-particle digestion is compared to an in-solution digestion and an in-gel digestion of the protein corona. Interestingly, a range of different protein classes were found to be recovered to greater or lesser extents among the different methods. Apolipoproteins were detected at lower concentrations when a surfactant was used to solubilize peptides, whereas immunoglobulins in general have a high affinity for nanomaterials, and thus show lower recovery using on-particle digestion. The optimized on-particle digestion was validated using 6 nanomaterials and proved capable of recovering in excess of 97% of the protein corona. These are important factors to consider when designing corona studies and modeling corona formation and impacts, highlighting the significance of a comprehensive validation of nanomaterial corona analysis methods.

## 1. Introduction

A decade ago, the term nanomaterial (NM) protein corona was coined to describe the layer of absorbed proteins acquired by NMs in contact with biological or environmental fluids [1,2]. As a result, a new area of research has grown to eminence in the field of analysis and characterization of this acquired coating of biomolecules [1,3,4]. Research to date has demonstrated that this corona plays a vital role in how cells “see” NMs, and as such, it facilitates cellular uptake and distribution, which, in turn, leads to altered toxicological profiles compared to pristine NMs [5,6]. Due to the vital function the corona performs in these processes, well-characterized methods for its isolation and analysis are paramount to ensuring the corona is correctly and thoroughly characterized, and to facilitate through understanding and confirmation that these processes are indeed a product of the corona and not due to other extrinsic properties.

To date, a broad range of corona isolation techniques have been utilized within the NMs community; however, very few have been properly characterized and validated in terms of their protein recovery, digestion efficiency and reproducibility. When the protein corona initially became popular, it was common to incubate the NM-corona complex in Sodium Dodecyl Sulfate polyacrylamide gel electrophoresis (SDS-PAGE) starting buffer to isolate the corona. The released proteins would then be separated using SDS-PAGE electrophoresis followed by excision of several individual protein bands of interest and their digestion and analysis using nano-liquid chromatography-mass spectrometry (nano-LC-MS) [4,7,8]. This method, however, has significant drawbacks—primarily that only a very small proportion of the proteins present in the corona are analyzed, as this approach is biased towards the most abundant proteins that are visible with a Coomassie Blue stain. To extend the quality and depth of proteins characterized in the corona, methods for global analysis of the corona have been implemented. These include precipitating proteins from the NM surface using commercial reagent kits [9] or using SDS-PAGE starting buffer incubations prior to the removal of SDS using commercial surfactant/detergent filters to prevent LC-MS fouling [10]. These protein isolation methods harbor the risk of losing proteins via incomplete precipitation and re-dissolution or as a result of sample losses via gel electrophoresis. An alternative method that mitigates these specific risks is to perform a tryptic digest on the intact NP-corona complex, a so-called on-particle digestion. This method has begun to acquire traction within the corona community [11,12], and by using fewer steps between formation of the NM-corona complex and the digested peptide sample being ready to analyze, there is less risk of introducing errors (e.g., loss of proteins), while also improving throughput. It is, however, also possible that this approach may allow the peptides to re-adsorb on the NM surface, something which has never been investigated in the studies currently reported in the literature, or that the NM-corona complex may prevent full access of trypsin to the cleavage sites thus impeding digestions of the adsorbed proteins. So far, there has been no attempt to determine which, if any, of these approaches provides the most unbiased and comprehensive characterization of the NM protein corona. Furthermore, without a full understanding of the relative performance of these methods it is difficult to further optimize them to improve protein coverage and provide a complete and reproducible description of NM coronas for correlation with signaling impacts. 

In recent years, there has been an increased interest in modeling of the formation of the protein corona and utilization of corona characteristics to predict NM cellular uptake and toxicological effects, via, for example, Quantitative Structure-Activity Relationships (QSARs) [13,14]. To build these models, high-quality data is required regarding the composition of NM coronas, and the cellular or organismal responses to the corona-coated NMs. For such models to be predictive, it is essential to obtain this data with properly characterized and validated sample preparation, corona isolation and analysis methods, a factor that has been overlooked to date. Thus, no effort has yet been made to determine if there are any differences in the corona compositions resulting from the different approaches applied to the same NM/biofluid mixtures, if corona isolation efficiency is dependent upon NM properties, or whether there are any biases towards different protein classes that need to be accounted for in these different approaches to determining the NM protein corona. It is only once this is known that comprehensive and precise models for predicting corona composition, and for correlating corona composition with uptake and impacts, can be constructed. Indeed, there are some contradictory reports in the literature regarding the utility of the corona for predicting NMs cellular uptake [15,16,17], which may be due to the specific NM/cell combinations studied to date, or could be a result of differences in the methodologies utilized to isolate the corona proteins. The current study aims to address this knowledge gap by performing a comparison of the existing methods.

While there are many analytical techniques that have been used to investigate the protein corona, including flow cytometry [18], nuclear magnetic resonance [19] and quartz crystal microbalances [20], these often require prior knowledge of protein present or cannot be used to identify proteins. However, hyphenated mass spectrometry techniques offer a global analysis of proteins and has the ability to identify these without pre-existing knowledge of the corona content, As such, it is common practice that once isolated, peptides are analyzed using nano-LC-MS, the workhorse of proteomic workflows [21,22]. This method is poorly suited for polar peptides that elute in the solvent front and for very large peptides that do not elute from the column, leading to lower sequence coverage or the lack of detection of small polar proteins. One technique that is underutilized in the proteomics field and has yet to be applied to NM corona analysis is capillary electrophoresis-mass spectrometry (CE-MS) [23]. CE-MS is an orthogonal/complementary separation approach to LC-MS [24,25,26,27] and can be found in some NM labs where they are used to characterize a range of NM properties including the absolute size, relative size, size distribution, composition, surface charge and zeta potential, in a single measurement [23]. CE-MS has been used for a wide range of proteomic studies [24,28,29,30,31], and due to its low solvent usage (nL/min), low sampling volume and aqueous solvents, there are significant green chemistry benefits to its application, which is particularly pertinent in the context of environmental nanoscience. Capillary Electrophoresis with Electro Spray Ionization (CESI-MS) is the latest development of CE-MS, using low nanoliter per minute flow rates without the diluting effect of a sheath liquid to enable highly sensitive analyses. Furthermore, this technique comes with an integrated electrospray ionization emitter thus mitigating any dead volume issues that arise in conventional CE-MS between the capillary and the ESI emitter, thus making the analysis more reproducible and minimizing instrument down time [32]. Thus, the method offers enormous potential also for NM-corona analysis.

The aim of this study is to introduce CESI-MS as a potent new tool for protein corona characterization, as such it is compared to the current gold standard analytical platform, nano-LC-MS. Furthermore, another goal of this study is to develop a robust and global protein corona isolation method that allows the comprehensive and accurate characterization of the complete NM protein corona, spanning the entire molecular weight range of proteins and from polar to non-polar peptides. The developed method for corona isolation is compared to other commonly used methods (in-solution, in-gel) to determine any biases in the analysis that can be accounted for in future modeling work, as well as by experimentalists when choosing which approach to apply in their research. 

## 2. Materials and Methods

### 2.1. Nanoparticle Characterization

DLS and Zeta-potential measurements were performed on a Malvern Zetasizer (nanoZS) (Malvern Panalytical, Malvern, UK). Colloidal silica nanoparticles (SiO_2_ NMs) Ludox TM-40, 40% *w/v*, (Sigma, Merck, Darmstadt, Germany), 100 nm PS NMs and carboxylated PS NMs (Polysciences Inc., Hirschberg an der Bergstrasse, Germany), uncapped, PVP and dispex AA4040 capped titanium dioxide (TiO_2_) NMs with a primary size of 13 nm (Promethean Particles Ltd., Nottingham, UK) were dispersed in deionized water (18.2 MΩ·cm at 25 °C) at a concentration of 4 mg/mL at pH 6.98. Samples were analyzed at 25 °C with an equilibration period of 2 min prior to the measurement beginning as stipulated previously [33]. For both DLS and Zeta potentials 4 samples were analyzed consecutively each composed of 3 technical replicates per sample. These data were collected and averaged to calculate, hydrodynamic diameter, polydispersity index (PDI), zeta potential and electrophoretic mobility.

### 2.2. Human Plasma

Blood was collected from a healthy 38-year-old male individual (in accordance with the ethical principles stated in the Helsinki declaration) in lithium-heparin-coated tubes (Sarstedt, Nümbrecht, Germany) to prevent blood coagulation. The samples were centrifuged for 15 min at 1500 g at 4 °C. The supernatant was collected, pooled and stored at −80 °C until use. 

### 2.3. Plasma Protein Corona Formation

Thawed plasma samples were centrifuged for 5 min at 4000 g to remove precipitated proteins. For protein corona formation, 1 mg of SiO_2_ NMs was incubated with 250 µL of plasma for 1 h at 37 °C, shaking at 300 rpm. NMs and their associated coronas were centrifuged for 5 min at 2000 g and the plasma supernatant was removed. The particles were washed once with 250 µL of PBS buffer to remove unbound proteins using conditions similar to plasma (osmolarity and ion concentration) and then twice with 250 µL of ammonium bicarbonate (ABC) buffer (100 mM, pH 8.0) (>99.5%, Thermo Scientific, Vienna, Austria), to remove nonvolatile salts and generate optimal conditions for Trypsin, by centrifuging for 5 min at 2000 g and removing the supernatant. NMs were transferred to a fresh vial after the second ABC wash.

### 2.4. On-Particle Protein Digestion

To reduce protein disulfide bonds, the NM-corona pellet was dissolved in 20 µL ABC buffer (100 mM, pH 8.0) containing 10 mM dithiothreitol (>99%, Roth, Karlsruche, Germany) and incubated for 30 min at 56 °C. For enzymatic digestion, 0.5 µg of trypsin (Sequencing grade, Promega, Mannheim, Germany), in 20 µL ABC buffer, was added, and the sample was incubated for 16 h at 37 °C. To enhance the digestion reaction, the sample was placed in an ultrasonic bath for the first hour of incubation. Cysteines were alkylated with 20 µL iodoacetamide (>99%, Sigma, Vienna, Austria), 55 mM in 100 mM ABC buffer, at room temperature for 20 min. The resulting peptides were ZipTip enriched using C18 Tips (100 µL, Pierce, Thermo Scientific, Vienna, Austria), lyophilized and stored at −20 °C for subsequent analysis. 

To increase solubility during enzymatic digestion the aforementioned method was modified in that 10 µL of ABC buffer containing either 0.1% *Rapigest* SF™ (Waters, Elstree, UK), 6 M urea (>99.5%, Merck, Darmstadt, Germany) or 0.1% *Rapigest* SF™ plus 6 M urea was added to the sample, to give a final concentration of 1.5 M, after the disulfide bond reduction step. To compensate for the increased volume, trypsin was dissolved in 10 µL instead of 20 µL. 

### 2.5. Sodium Dodecyl Sulfate Polyacrylamide Gel Electrophoresis (SDS-PAGE)

Sodium Dodecyl Sulfate polyacrylamide gel electrophoresis (SDS PAGE) was performed according to standard procedures [34]. In brief, corona-coated NMs were incubated in SDS sample buffer for 5 min at 95 °C, the sample was centrifuged at 10,000 g for 10 min and 10 µL of solubilized proteins (in sample buffer: 250 mM Tris, 10% SDS, 30% (*v:v*) glycerol, 10 mM DTT, 0.05 (*w/v*) Bromophenol blue) were loaded onto a 4–20% Tris-Glycine gel (Novex, Thermo Scientific, Vienna, Austria), along with a protein ladder to estimate protein sizes (protein standard range: 11–245 kDa, New England Biolabs GmbH, Frankfurt, Germany). Proteins were separated using a 25 mM Tris, 190 mM glycine, 0.1% *w/v* SDS running buffer at pH 8.6. Proteins were stained using Coomassie brilliant blue R−250 and in-gel protein digested, whereby the area that contained proteins (~1 cm², determined by Coomassie blue staining) was excised from the one-dimensional gel, cut into small pieces and transferred into a 0.5 mL vial. Gel pieces were washed twice with ABC buffer (100 mM, pH 8.0), twice with ABC buffer/Acetonitrile (1:1), and dried in a vacuum centrifuge. To reduce the disulfide bonds within the proteins, 160 µL of 10 mM DTT in ABC buffer was added to the gel pieces followed by incubation at 56°C for 30 min. Gel pieces were centrifuged, the supernatant removed, and 160 µL acetonitrile was added. After 10 min of incubation, acetonitrile was replaced by 160 µL of 55 mM iodoacetamide in ABC buffer. After 20 min of incubation at room temperature in the dark, the gel pieces were washed with ABC buffer for 5 min and shrunken by adding 150 µL of acetonitrile for 15 min. After removing the supernatant, the gel pieces were again dried in a vacuum centrifuge. The gel pieces were then swollen in 200 µL of 10 mM ammonium bicarbonate buffer containing 2 µg of trypsin (Sequencing grade, Promega). For enzymatic digestion, the samples were incubated for 16 h at 37 °C. Peptides were thereafter extracted by adding 160 μL of acetonitrile/5% formic acid (2:1) to the gel pieces and incubating at 37 °C for 15 min. After collecting the supernatant, the gel extraction was repeated with 160 µL of acetonitrile/5% formic acid (2:1), isopropanol/2% formic acid (1:1), and with acetonitrile (each time incubating at 37 °C for 15 min). The supernatants were combined, lyophilized, and stored at −20 °C for subsequent analysis [35]. 

### 2.6. In-Solution Digestion

For in-solution digestion, proteins bound to the NMs were incubated for 5 min at 95 °C in 25 µL of 8 M Urea, 2% *w/v* CHAPS (3-[(3-Cholamidopropyl)dimethylammonio]-1-propanesulfonate hydrate) (>98%, Roth) [34]. The sample was centrifuged for 5 min at 20,000 g, and the proteins present in the supernatant were precipitated with 125 µL of acetone. Supernatant was removed and the NM-corona pellet was washed, as described above, and proteins were thereafter re-dissolved in 20 µL ABC buffer containing 10 mM dithiothreitol. All further steps were according to the procedure described for the on-particle protein digestion section.

### 2.7. Capillary Electrophoresis with Electro Spray Ionization Mass Spectrometry (CESI-MS) Analysis

For CESI-MS analysis of peptides a CESI 8000 Plus capillary electrophoresis system (Sciex, Brea, CA, USA) was coupled to a Q-Exactive HF mass spectrometer (Thermo Scientific, Bremen, Germany). Capillary electrophoresis was performed on a neutral capillary (total length: 90 cm, i.d.: 30 µm, provided by Sciex), which served as the separation capillary as well as an electrospray emitter. The analyses were performed as described previously [31]. In brief, capillaries were rinsed with acetic acid (>99.8%, Sigma, Vienna, Austria), 100 mM, pH 2.9, which was also used as the background electrolyte (BGE). Samples were injected hydrodynamically by applying a pressure of 5 psi for 10 s. The separation was performed at +30 kV with a pressure gradient applied. The gradient profile was: 0–10 min, 1 psi; 10–35 min, 1.5 psi and 35–45 min, 5 psi. The Q-Exactive HF mass spectrometer was operating in data-dependent acquisition mode. Full scan spectra were acquired with a scan range of *m/z* = 250–2000 and a resolution of 60,000 (at *m/z* = 200). The 10 most intense precursor ions were selected for HCD fragmentation. The MS/MS spectra were acquired at a resolution of 30,000 with a normalized collision energy of 28. All other instrument settings were as described elsewhere [24,36]. All experiments were performed in triplicate, technical replicates were also performed in triplicate where described.

### 2.8. Data Analysis

The MS data files were processed using Proteome Discoverer version 2.2 (Thermo Scientific, Bremen, Germany). MS/MS spectra were searched against the Uniprot human reference proteome database (20,939 entries, last modified 2 February 2018) using the Sequest HT search engine. Peptide identification settings were: Enzyme for protein cleavage—Trypsin; two missed cleavages were allowed. Fixed modification was carbamidomethyl-cysteine; variable modifications were oxidation of methionine and acetylation of the protein N-terminus. Precursor mass tolerance was set to 10 ppm; fragment mass tolerance was 20 mmu. Maximum false discovery rate (FDR) for protein and peptide identification was set to 1%. For label-free quantification, the Minora Feature Detector node was set to high-confidence PSM (peptide spectrum matches) only, with at least two isotopic peaks present in the isotope pattern. Proteins were quantified as the sum of peptide peak intensities. Abundance normalization (based on total peptide amount) and ratio calculation of peptides and proteins were conducted in Microsoft Excel. For statistical analysis p-values were calculated using paired Student’s *t*-tests with subsequent Benjamini–Hochberg correction using a maximum FDR set to 0.05.

## 3. Results and Discussion

### 3.1. Particle Characterization

Dynamic light scattering (DLS) analysis of the Ludox TM-40 SiO_2_ NMs in MilliQ water revealed an average hydrodynamic radius of 34.8 ± 0.6 nm with a polydispersity index (PDI) of 0.15 ± 0.01. In addition, the zeta potential was determined to be −42.0 ± 1.7 mV on average, with an electrophoretic mobility of −3.3 ± 0.1 µmcm/Vs. The two polystyrene NMs had an average hydrodynamic diameter of 106.9 ± 1.7 nm and 111.2 ± 2.6 nm for the uncapped and the carboxylated PSNMs respectively, both with PDIs of 0.04 ± 0.001. Their respective zeta potentials are −61.2 ± 2.7 mV and −63.4 ± 2.5 mV on average, with electrophoretic mobilities of −4.8 ± 0.2 µmcm/Vs and −5.0 ± 0.2 µmcm/Vs. The titanium dioxide (TiO_2_) NMs were much more polydisperse as a result of the anatase structure and they fact that they easily agglomerate. The measured average hydrodynamic diameters were 922.4 ± 81.3, 1757 ± 793.4 and 2076.5 ± 666.7 nm with PDIs of 0.06 ± 0.005, 0.27 ± 0.15 and 0.58 ± 0.32 for the uncapped, dispex and PVP capped particles, respectively. Their respective zeta potentials were 24.3 ± 1.3 mV, 24.9 ± 1.5 mV and 8.3 ± 1.1 mV with electrophoretic mobilities of 1.90 ± 0.1 µmcm/Vs, 1.95 ± 0.1 µmcm/Vs and 0.65 ± 0.1 µmcm/Vs. All characterization data can be found in Supplementary Materials SI-1.

### 3.2. On-Particle Digestion with Subsequent CESI-MS Analysis

Following corona formation, the unbound proteins were washed away using a 3-step wash, which has been shown to be effective in previous work [7]; this approach was selected over others, such as the glucose cushion [9], as it minimizes the number of steps and sources of contamination or sample loss. Subsequent to this, the on-particle digestion was performed all within the same reaction tube, thereby minimizing any potential loss of proteins or peptides. This process started with the reduction of disulfide bonds prior to enzymatic cleavage of proteins with 0.5 µg of trypsin overnight, and was completed with an alkylation step resulting in formation of carbamidomethyl-cysteines to circumvent crosslinking of peptides. Since CE separation could be influenced by accompanying components in the sample, e.g., salts or detergents, to enhance sample quality a ZipTip enrichment, a reproducible and high-recovery approach to purify and concentrate peptides was performed prior to CESI-MS analysis. 

**Figure 1:**
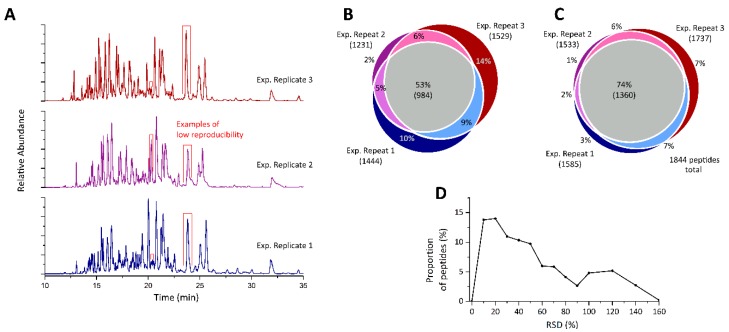
Analysis of SiO_2_ NM protein corona enzymatically digested on-particle using trypsin in the presence of 100 mM ammonium bicarbonate (pH 8.0). (**A**) Base peak electropherograms of experimental replicates, red boxes indicate visual discrepancies between replicates. Venn diagrams of peptides (**B**) identified via MSMS and (**C**) detected as precursor mass in the three experimental replicates. (**D**) Reproducibility of peptide quantifications shown as a histogram of relative standard deviations (RSDs) calculated for each individual peptide.

The initial characterization of the SiO_2_ NM corona, using 3 experimental replicates (complete workflow from NM exposure to plasma until CESI-MS analysis), resulted in a total of 1844 peptides being identified, corresponding to 121 high-confidence protein identifications (Supplementary Materials SI-2). A critical visual assessment of the electropherograms, shown in Figure 1A, revealed discrepancies between the experimental replicates. This can be seen clearly in the relative intensities of the two peaks migrating at 24 min or the peak at 20 min, which differ significantly. When investigating the overlap of peptides identified in the replicates, it became apparent that just 53% of the peptides could be identified in all replicates, whereas 26% could be identified in a single replicate only (Figure 1B). As the MS instrument was operated in data-dependent mode, some of the peptides identified in a single analysis only may not have been selected for MS/MS fragmentation for subsequent replicates. This is a known pitfall of label-free quantification [9], which is of particular concern, as protein coronas are typically thought to vary quantitatively rather than qualitatively in protein content, between particle physical and chemical properties [34]. To attenuate the effect of varying precursor selection between replicates, the peptides corresponding to precursor ions were searched for via a label free data analysis using Proteome Discoverer software. This was achieved by performing a migration time alignment and quantification of peptides based on their precursor intensity, as they were identified in each replicate. This increased the number of peptides found in all analyses from 984 to 1360, which corresponds to 74% of all peptides, whereas the number of single peptide quantifications was reduced by 15% (Figure 1C). Since quantitative values for most peptides had been determined, a statistical analysis was carried out for further evaluation of experimental reproducibility. The relative standard deviations (RSDs) of the signal intensity for each peptide was determined and plotted (Figure 1D). As seen in Figure 1D, RSD values for peptide quantification ranged from 0 to 160%. Approximately 14% of all peptides could be quantified with RSDs of ≤10% with a further 14% having ≤20% RSD. However, 32% of all peptides returned an RSD of ≥50%, making them unsuitable for quantification.

**Figure 2:**
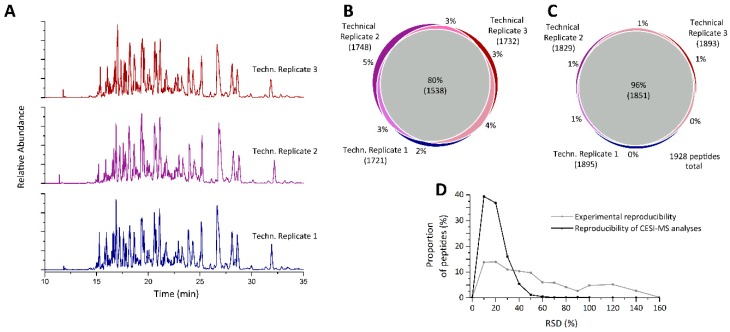
Technical reproducibility of CESI-MS analyses when analyzing a digested SiO_2_ NM protein corona. (**A**) Base peak electropherograms of technical replicates, Venn diagrams of peptides (**B**) identified via MSMS and (**C**) detected as precursor mass in the three technical replicates. (**D**) Reproducibility of peptide quantifications shown as histogram of relative standard deviations.

**Figure 3:**
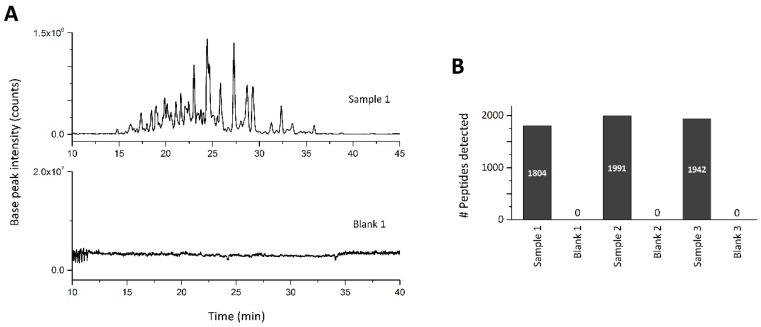
Carryover analysis of CESI-MS when analyzing a digested SiO_2_ NM protein corona followed by a water blank. (**A**) Base peak electropherograms of SiO_2_ NM protein corona above base peak electropherogram of subsequent blank injection. (**B**) Peptides identified in each sample injection and subsequent blank.

Due to the relatively poor reproducibility, additional development and validation was performed to enhance the quality of the corona characterization, starting with the analytical step. Initially, the reproducibility of the CESI-MS analysis was evaluated by analyzing an equimolar mixture of the three technical replicate samples (same sample injected multiple times). This was analyzed in triplicate and resulted in the detection of 1928 peptides (Supplementary Materials SI-3). Corresponding electropherograms can be seen in Figure 2A. The overlap in peptides detected in the three replicates was much greater than previously, with 80% identified using MS/MS (Figure 2B) and 96% of peptides detected by their precursor ion alone (Figure 2C). Only 23 peptides, equating to a little over 1% of all peptides, were detected in a single analysis only. In addition, the reproducibility of peptide quantification was significantly enhanced. Almost 40% of the peptides could be quantified with a relative standard deviation ≤10% and <2% showed RSDs greater than 50% (Figure 2D). This high level of reproducibility is in line with previously published quantitative results obtained when analyzing single peptides [37,38]. The reproducibility of the CESI separation was found to be excellent, with mean and median migration time RSDs of 0.302% and 0.045%, respectively. Finally, a carryover analysis was performed in triplicate by injecting the pooled sample followed by an injection of water using the same capillary and background electrolyte. This resulted in no peptides being detected at all in the blank samples (see Figure 3A,B), meaning that no peptides were retained within the capillary from the previous sample injection. This ensures a high degree of confidence that peptides detected in any one run are indeed from the sample of interest, thus enabling the short CESI method (<50 min) to be applied, as no cleanup method is required. To compare the CESI-MS recovery and quantification data to that from the most popular method utilized in the corona literature to date, we also analyzed these samples by nano-LC-MS (Supplementary Materials SI-4 and SI-5). We found that, while nano-LC-MS identified more proteins and peptides overall, the top 20 proteins did not change between CESI-MS and nano-LC-MS. However, the CESI-MS proved to be slightly more reproducible in terms of peak area and migration time of peptides than nano-LC-MS (Supplementary Materials SI-5). In addition, unlike CESI-MS, a larger amount of carryover was observed between nano-LC-MS injections, which required 2 blank injections after each sample to remove them fully, thus greatly reducing the throughput of analysis, with an injection to injection time of 50 min for the CESI-MS compared to 3 h for nano-LC-MS [39]. Due to the excellent CESI-MS reproducibility, it is apparent that the on-particle digestion corona isolation method is responsible for the poor reproducibility evident in Figure 1. Optimization of the digestion and isolation steps are presented below.

### 3.3. Optimization of Enzymatic On-Particle Digestion

**Figure 4:**
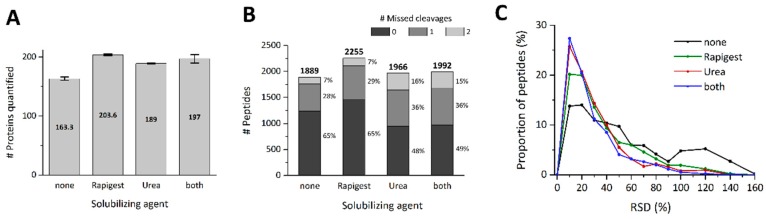
Impact of solubilizing agents during on-particle digestion of the SiO_2_ NM corona on the total numbers of (**A**) proteins and (**B**) peptides identified in three experimental replicates, and (**C**) on the reproducibility in peptide quantification within triplicate experiments.

A previously published study investigating the impact of different surfactants on enzymatic digestion found that their use can enhance the peptide identification rate significantly [40]. Therefore, in addition to improving reproducibility, modifications were made to the method to enhance the number of peptides generated in order to obtain a more comprehensive description of the protein corona. As such, two agents, urea and *Rapigest* SF™, were investigated. Urea is chaotropic and is commonly used at 8 M to weaken hydrophobic interactions within proteins leading to partial unfolding. However, 8 M of urea would reduce the digestion efficiency of trypsin; therefore, a concentration of 1.5 M was used. Urea was added following the reduction step to keep artificial carbamylation of lysines and protein N-termini to a minimum [41]. The second solubilizing agent investigated was 0.1% *w/v Rapigest* SF™, a mild anionic detergent added during the digestion process. However, if not depleted, surfactants can adhere to the capillary surface changing the electroosmotic flow, and high concentrations of salts can interfere with sample stacking and thus result in peak broadening. As such, *Rapigest* SF™ was cleaved at a low pH and the resulting fragments removed [40]. Urea is also easily removable prior to MS analysis via C18 ZipTip enrichment. To evaluate the influence of the two added reagents, triplicate analysis was performed for 4 different conditions: pure 100 mM ammonium bicarbonate (ABC) buffer, ABC buffer with 1.5 M urea, ABC Buffer with 0.1% *w/v Rapigest* SF™, and ABC buffer with a combination of both 1.5 M urea and 0.1% *w/v Rapigest* SF™. To obtain the most comparable results, all sample preparation steps were identical except the addition of the solubilizing agents. A database search of the acquired MS data revealed that *Rapigest* SF™ enabled the detection of the greatest number of peptides and proteins, with 2255 (Figure 4B) and 196 (Supplementary Materials SI-6), respectively. This is an increase in protein and peptide yield of 7.7% and 13.2%, respectively, compared to the next best approach, combined *Rapigest* SF™ and urea, which resulted in 182 proteins and 1992 high-confidence peptides being identified. Full details of the proteins and peptides identified under the 4 (urea and *Rapigest* SF™) conditions tested can be found in Supplementary Materials SI-6.

Interestingly, when examining the characteristics of the identified peptides, it was found that the addition of urea increases the occurrence of missed cleaved peptides significantly (Figure 4B). Samples without urea contained, on average, 65% fully cleaved peptides, and 29% with one and 7% with two missed cleavage sites, whereas samples treated with urea contained only 48% fully cleaved peptides, but 36% and 16% peptides with one and two missed cleavage sites, respectively. Additional evaluation of the influence of these reagents on peptide quantification demonstrated that the addition of both agents improved reproducibility. The best result was obtained when combining the two agents, with 48% of all peptides showing an RSD below 20%. The number of peptides with an RSD above 50% was reduced significantly from 32% using no additive to 10% when combining urea and *Rapigest* SF™ (Figure 4C). Due to the potential to radically alter migration time characteristics by the addition of both a salt and a surfactant, the migration time reproducibility was assessed. The analysis revealed a mean and median migration time RSD of 0.350% and 0.052%, respectively. This is remarkably similar to the analytical replicates, highlighting the highly reliable nature of this methodology, ensuring a high degree of confidence in protein identification and quantification. Since the addition of any of the tested surfactants improves the reproducibility of peptide quantification, we consider their use to be generally advisable. Nevertheless, when comparing the improved reproducibility values to the technical reproducibility of the CESI-MS analysis (Figure 2D), the reduction in reproducibility related to the sample preparation (digestion and isolation) is still remarkable and reveals the importance of performing experimental replicates for reliable protein quantification. 

**Figure 5:**
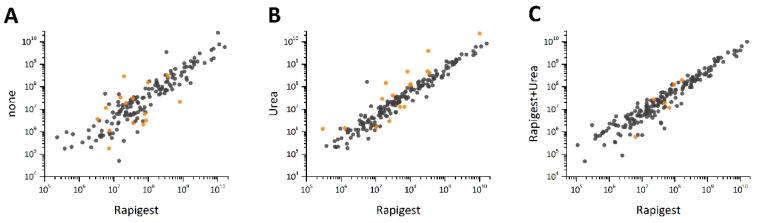
Pair-wise comparisons of protein abundances in SiO_2_ NM corona depending on the solubilizing agent used, (**A**) *Rapigest* SF™ treated versus untreated samples, (**B**) *Rapigest* SF™ versus urea treated samples, (**C**) *Rapigest* SF™ versus *Rapigest* SF™ plus urea treated samples. Proteins labeled in orange show a statistically significant difference in abundance between the two experimental triplicates with a ratio of at least 2.

Based on these results, the *Rapigest* SF™ approach was considered the best in terms of the number of proteins and peptides identified combined with the favorable digestion efficiency observed. To evaluate this approach for accurately describing the NM protein corona, a comparison of protein abundances obtained by the different approaches was performed. The goal was to investigate whether proteins were lost or were less abundant when using *Rapigest* SF™ alone, in combination with urea, or urea alone. Pairwise comparisons of the different approaches were conducted, in addition to a Student’s t-test with a Benjamini–Hochberg FDR test for each protein quantified (for details see Supplementary Materials SI-7). In addition, pair-wise scatter plots were constructed to compare protein abundances graphically (Figure 5). The proteins found to be statistically different between treatments are detailed in Table 1. Protein abundances do not always correlate well between the different method optimizations (Figure 5A–C); this was particularly evident for the comparison of *Rapigest* SF™ with the pure ABC solution (Figure 5A). The poor reproducibility in peptide abundances of the ABC-only approach resulted in deviating protein abundances within the triplicate experiments; therefore, abundance differences, if present, were rarely statistically significant. Much better overall correlation was observed between the other approaches (Figure 5B,C), with the best correlation observed between the *Rapigest* SF™ and the combined solubilizing reagent method. When comparing the urea to the *Rapigest* SF™ approach, 12 proteins were found to be significantly reduced in the *Rapigest* SF™ samples (Figure 5B). Interestingly, 5 out of these 12 proteins were Apolipoproteins (Table 1), which are frequently reported in NM protein coronas and play important roles in transport of NMs (into cells and across biological barriers) [42,43,44]. A recent study found that Apolipoprotein A-I showed an increased abundance when incubating NMs with plasma in the presence of surfactants. The reason suggested for this was a lower propensity of Apo A-I to denature compared to other proteins, thereby altering the composition of the protein corona significantly [45]. This finding might explain the *Rapigest* SF™ results; because apolipoproteins are less prone to detergents, other corona proteins might get digested with higher efficiency relative to apolipoproteins, resulting in a seemingly reduced abundance of apolipoproteins in the *Rapigest* SF™ treated samples.

### 3.4. Evaluation of Protein Corona Isolation Efficiency

**Figure 6:**
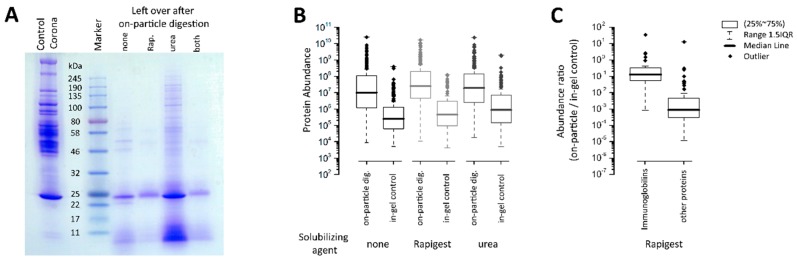
Analysis of the left-over proteins after on-particle digestion of corona on SiO_2_ NMs. (**A**) SDS-PAGE of analytes bound onto the SiO_2_ NMs after on-particle digestion. (**B**) Box plot illustrating protein abundances determined for each individual protein in the original on-particle digestion and in the in-gel control. (**C**) Calculated protein abundance ratios (on-particle/in-gel control) revealed significantly higher ratios for immunoglobulins remaining attached to the NMs compared to other proteins.

To evaluate the recovery of on-particle enzymatic digestion, the SiO_2_ NMs were washed with 100 mM ABC buffer to remove free peptides and incubated with SDS sample buffer to dissolve potential undigested proteins. These proteins were thereafter separated by SDS-PAGE, in-gel digested, and the resulting peptides were analyzed by CESI-MS. When separating these incompletely digested proteins by SDS-PAGE, it became apparent that nearly all proteins had been completely digested compared to the complete SiO_2_ NM protein corona, which was also separated by SDS-PAGE as a control (Figure 6A). Only a single protein near the 25 kDa marker protein was visible after on-particle digestion with *Rapigest* SF™, in addition to some small peptides near the bottom of the gel. As seen in the gel, the protein present in the *Rapigest* SF™ samples at 25 kDa seems to exhibit a slightly higher mass compared to the protein present in the samples with urea and without surfactant, thereby indicating that these proteins might have different identities. Subsequent CESI-MS analysis indeed revealed that the highest abundant protein was Apolipoprotein A-I in the samples when no solubilizing agent or urea was added, whereas immunoglobulin light chain was the highest abundant protein in the sample containing *Rapigest* SF™ (see Supplementary Materials SI-8). 

To better understand the digestion efficiency and the ability to keep the resulting peptides suspended, similar sample volumes to the original on-particle digests and their corresponding in-gel controls were analyzed by CESI-MS and the intensities of detected proteins compared. Since the in-gel control samples contained all analytes that remained on, or (re-)adsorbed to, the SiO_2_ NMs, it was not possible to distinguish between previously whole proteins and incompletely digested proteins or peptides. Therefore, it is best to consider the MS result to be a useful method to predict sample losses due to incomplete solubilization of peptides. Although many proteins can be identified in the in-gel controls (e.g., 80 proteins with high confidence in the *Rapigest* SF™ control), their abundance is extremely low. For example, proteins detected in the *Rapigest* SF™ in-gel control showed a total signal intensity of 8.5 × 10^8^ counts, which is a reduction of 138-fold compared to the original on-particle digestion with 1.17 × 10^11^ counts (see Supplementary Materials SI-8). Figure 6B illustrates the distribution of protein abundances determined for each individual protein in each sample type. When calculating abundance ratios for these individual proteins, a median ratio of 0.005 was observed between the digested proteins and the proteins re-adsorbed onto the NM surface. This means that, on average, only 0.5% of each protein or resulting peptides were re-adsorbed onto the NMs following on-particle digestion and subsequently not analyzed. This proportion was slightly higher, at 0.9%, when not using a solubilizing agent and, interestingly, even higher in the sample containing urea, where 3.3% of digested proteins were re-adsorbed onto the NM surface (Figure 6B and Supplementary Materials SI-8 for calculations). Based on these results, on-particle digestion in the presence of *Rapigest* SF™ was considered to be the most suitable sample preparation method for protein corona analysis. 

A closer inspection of individual protein ratios revealed that the ratios were not normally distributed. Immunoglobulins were found to have significantly higher ratios compared to all other proteins. The median ratio (abundances determined for each individual protein in the original on-particle digestion and in the in-gel control) for immunoglobulins was 0.12, whereas for all other proteins, a median ratio of 0.0009 was found. This effect was found in all samples, independent of the solubilizing agent used (Figure 6C), indicating that immunoglobulins or peptides thereof have higher affinity to the NMs than other proteins. This may be of significance when it comes to modeling the formation of the protein corona and correlating corona with cellular uptake and impacts, and is possibly a property that to date has gone unnoticed.

### 3.5. Comparison of On-Particle Digestion to Other Sample Preparation (Corona Isolation) Techniques

To benchmark this novel proteomics approach, combining on-particle digestion in the presence of *Rapigest* SF with CESI-MS analysis, the same NM coronas were analyzed using two common approaches that are well documented in the literature. The first is a modification of the SDS-PAGE protein band excision and in-gel digest, as this restricts the analysis to a limited number of proteins, so the method was modified to enable a global digest of the protein corona. The second procedure consists of corona solubilization using urea and CHAPS, followed by protein precipitation, and in-solution digestion. This latter method is particularly common when using commercial protein precipitation kits.

**Figure 7:**
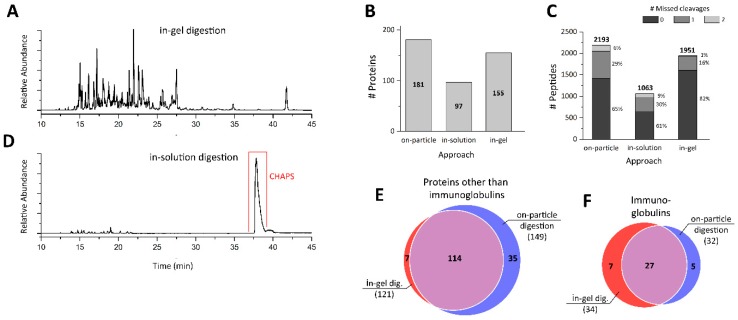
Analysis of SiO_2_ NMs protein corona using an in-gel digestion and an in-solution digestion-based approach. Base peak electropherograms of protein corona digested (**A**) in-gel and (**D**) in-solution. The impact of the different sample preparation (corona isolation) procedures on the total numbers of (**B**) proteins and (**C**) peptides identified. Venn diagrams of non-immunoglobulins (**E**) and immunoglobulins (**F**) identified by the on-particle and in-gel approach. All experiments were performed in triplicate.

To separate proteins by SDS-PAGE, the NMs were incubated in sample buffer, solid particles were removed by centrifugation, and the resulting solubilized proteins were loaded onto the gel. The protein containing area of the gel was thereafter cut and subjected to in-gel digestion. To minimize this area, the proteins were not fully separated, instead separation was stopped when the proteins had migrated about 2 cm into the gel. Peptides resulting from in-gel digestion were then Zip-Tip enriched and analyzed by CESI-MS (Figure 7A), resulting in 155 proteins (Figure 7B) and 1951 peptides (Figure 7C). Compared to the on-particle approach, this was a reduction of 14% for identified proteins and 11% fewer peptides. Interestingly, the proportion of fully cleaved peptides was 82%, and was much higher in the in-gel digested sample compared to the on-particle digestion, where only 65% were fully cleaved. The higher number of specifically cleaved peptides indicates that in-gel digestion enables a more efficient digestion than the on-particle digestion, which is not unexpected, given that cleavage sites may be hidden due to the orientation of proteins at the NM surface. This improved digestion efficiency in-gel is possibly a result of the proteins being linearized by SDS and not refolding into their native state; therefore, cleavage sites might be more accessible to trypsin. Nevertheless, the in-gel approach resulted in fewer proteins and peptides being detected and identified, suggesting loss of proteins and peptides throughout the steps of the isolation process. 

When comparing the protein identities, several immunoglobulins were identified with very low numbers of peptide spectrum matches. As these proteins occurred frequently in a single approach only, it may be that the database software had problems grouping them properly because of the limited number of unique peptides detected. To compare protein identities, proteins were split into two categories: immunoglobulins and non-immunoglobulins. In terms of non-immunoglobulins, 7 proteins were identified solely by the in-gel approach, whereas 35 proteins were exclusively identified by the on-particle approach (Figure 7E). Moreover, 6 out of the 7 in-gel proteins were found to be keratins, possibly introduced during in-gel digestion and originally not part of the protein corona. In terms of immunoglobulins, both approaches identified similar numbers of proteins, with 7 proteins unique to the in-gel approach and 5 unique to the on-particle approach (Figure 7F). In total, this result reflects the much higher sensitivity of the on-particle approach, which, along with the much shorter sample preparation time (1 day versus 3 days) and the lower scope for introduction of non-plasma proteins, are the main advantages that the novel approach presented here offers. Further information on proteins identified using in-gel digestion can be found in the Supplementary Materials SI-9.

A similarly short sample preparation time can be achieved using the second approach, which was compared to the on-particle approach. This approach is based on the solubilization of the protein corona using a buffer containing CHAPS and urea. Due to the incompatibility of their high concentrations with the enzymatic digestion step, the solubilized proteins were precipitated and re-dissolved in a digestion-compatible buffer. As such, acetone was used to precipitate the proteins, which were then dissolved in the same buffer used for the on-particle digestion (ABC buffer). After in-solution digestion, the resulting peptides were again Zip-Tip enriched and analyzed by CESI-MS. It is clear that in the electropherogram CHAPS is still present in the sample, even after peptide enrichment with Zip-Tips (Figure 7D). This hampered the analysis in two ways: (i) CHAPS binds to the C18 material lowering the peptide binding capacity, and thus significantly reduces the amount of peptides in the enriched sample; and (ii) it binds to the capillary surface during CESI-MS analysis, degrading the separation, and preventing reproducible analysis. Due to these limitations, only 97 proteins and 1063 peptides could be identified in triplicate experiments. The proportion of fully cleaved peptides was also reduced to 61% with respect to the on-particle approach. Due to this poor outcome, this approach was not investigated any further. This approach is more appropriate when combined with nano-LC-MS analysis, as CHAPS will elute during the trapping process and will thus have minimal effect on the nano-LC-MS data. 

This new on-particle digestion with CESI-MS analysis approach was used to characterize the composition of the SiO_2_ NM protein corona. To achieve this, a label-free quantification method was applied that has been extensively described in the literature; in brief, it utilizes the three most abundant peptides per protein to estimate protein quantity to allow comparison between samples [34]. Initially, the average intensity of each peptide was determined in triplicate experiments, before averaging the intensity of the three most abundant peptides per protein to calculate individual protein quantities and, later, the corona composition. Since plasma proteins might be extensively processed, e.g., via the attachment of post translational modifications (PTMs) such as glycans or via cleavages, their actual masses are unknown. We therefore decided to calculate the protein composition in ppm. A detailed list of quantified proteins can be found in the Supplementary Materials SI-10.

**Figure 8:**
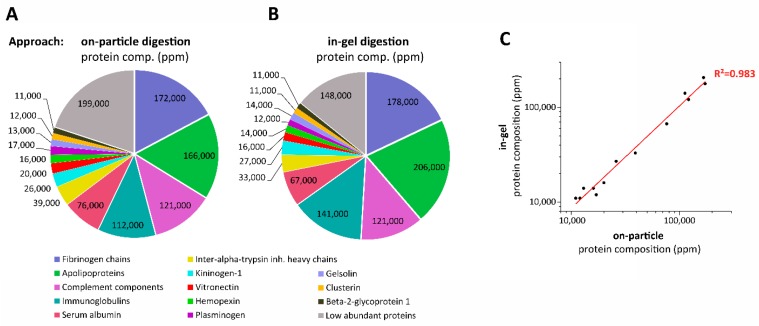
Protein composition in ppm from SiO_2_ NM coronas as determined either by (**A**) on-particle digestion combined with CESI-MS analysis, or (**B**) in-gel digestion combined with CESI-MS analysis. (**C**) Relationship between protein compositions calculated for the most abundant proteins (>10,000 ppm) in both approaches.

Analysis of the on-particle digestion reveals that the most abundant proteins were located within the group of fibrinogens, accounting for 172,000 ppm, followed by apolipoproteins with 166,000 ppm, complement components at 121,000 ppm, and immunoglobulins at 112,000 ppm (Figure 8A). Interestingly, in the in-gel digest, these protein groups appear to be present at a higher abundance, with 178,000 ppm for fibrinogens, 206,000, 121,000 and 141,000 ppm, respectively, for apolipoproteins, complement components and immunoglobulins (Figure 8B). This remarkable difference is caused by the fact that the on-particle approach is more sensitive and enables the quantification of a greater number of low abundant proteins, thus altering the apparent corona composition. The group of low-abundance proteins accounts for 199,000 ppm in the on-particle corona isolate, compared to 148,000 ppm in the gel-based approach. As can be seen in Figure 8C, the correlation in abundance of the most abundant proteins (>10,000 ppm) in the two approaches is very high (with R^2^ = 0.983), but nevertheless, if the approach utilized is not sensitive enough, one would overestimate the amount of the high abundant proteins, whereas low-abundance proteins would have been overlooked. 

### 3.6. Method Validation and Comparison of Protein Coronas on NMs of Different Compositions 

This newly developed CESI-MS method for determination of NM protein corona compositions was validated by isolating and characterizing the corona of a further 5 NMs of different composition and surface characteristics; these were carboxylated and non-carboxylated 100 nm polystyrene NMs, bare, polyvinylpyrrolidone (PVP) capped and Dispex AA4040 capped titanium dioxide (TiO_2_) NMs with a primary size of 13 nm (for DLS and Zeta potential data see Supplementary Materials SI-1). These NMs represent a range of chemistries, and increase in the complexity of their surface characteristics from basic carboxylation of spherical particles to large polymer-coated non-uniform anatase metal oxide NM aggregates. Furthermore, these NMs represent two major NMs to which humans and the environment are exposed, titania is found in many food stuffs, paints and cosmetics [46], whereas polystyrene NMs are commonly used as a surrogate for micro and nanoplastics, which are now ubiquitous in the environment [5]. The only variation to the method was that 0.5 mg of each NM was exposed to 250 µL of plasma; this gives the same concentration as that used for the development of the method, but scaled down to maintain use of the same plasma batch.

One of the keys points of this new approach is the high degree of protein recovery from the corona that was observed with the silica NMs. To ensure that this is not a result of the relatively simple nature of these particles, the recovery was assessed with an additional 5 NMs, which varied in complexity with surface modifications and more complex NM structure. Recovery was assessed by determining the total mass spectral signal of the on-particle digestion versus the in-gel digestion of any remaining proteins on the particle, the number of high-confidence protein identities in both digests, and the abundance ratios of proteins in both digests using the top 3 peptide abundances. As seen in Table 2, the recoveries determined using these 3 approaches were very high: in terms of mass spectra signal intensity and protein abundance ratios, the recovery exceeded 99%. As for the recovery of high confidence proteins, the TiO_2_ NMs all returned a 100% recovery rate, while the polystyrene NMs exceeded 96% recovery (Supplementary Materials SI-11). It is, however, important to state that high-confidence proteins identified in the in-gel digest of the remaining NMs following on-particle digest, albumin and apolipoprotein C-III on the plain PS and albumin, vitronectin and lysozyme C for the carboxylated PS NMs, were all found in much higher concentrations in the on-particle digest solution. This suggests that while a small proportion of these proteins or their corresponding peptides remained adsorbed on the NMs, a much greater proportion was recovered during the on-particle digestion. These results do, however, suggest that previous attempts to quantify the proteins in the corona are likely to have misrepresented the corona composition by not accounting for proteins that remained attached to the NMs following the extraction procedure, not investigating protein recovery, and not anticipating variations in protein recovery between NM species. As such, for true quantitative analysis of the protein corona, it is recommended that recovery studies of proteins be performed for all NMs being investigated in order to account for any that remain bound.

The final step of method validation was to characterize the protein coronas across all 6 NMs investigated as a proof of principal to demonstrate that the developed isolation and analysis methods are sensitive enough to detect variations in the corona composition arising from variation in NM composition and surface modifications. Here, protein abundances were summed based upon their 3 most abundant peptides to determine total protein content, and then the top 19 protein groups were expressed as a percentage of the total protein content, with a final group comprised of all other proteins present (Table 3).

It is clear that across the 6 NMs investigated, there are differential protein binding preferences between particles. Interestingly, serum albumin, which is generally regarded as one of the most abundant proteins in the corona, shows a large degree of variation among the different NMs. In the silica and polystyrene NMs, albumin is in the top 5 proteins, with the bare PS NMs adsorbing the greatest proportion of albumin; indeed, it is the only time a single protein makes up over 50% of all protein in the corona. By contrast, the TiO_2_ NMs protein coronas are made up of less than 1% serum albumin and apolipoproteins, which is a significant observation as the latter protein family is thought to play a vital role in NM uptake and distribution in cells and the body, with the potential for specific members to be used to facilitate NMs to cross the blood–brain (Apo-E) and blood–fetus barriers [42,44,47]. Interestingly, the PVP surface modification on the TiO_2_ NMs had a relatively neutral effect on the protein corona composition despite different sizes and zeta potentials. In contrast, the polyacrylic modification of dispex resulted in greatly reduced levels of fibrinogens in the corona, but increased proportions of prothrombin and alpha-2-HS-glycopeptide. The carboxylation of the PS NMs also had a large impact upon the corona composition, with albumin dropping from 57.7% down to just 16.9% of the total protein content, whereas vitronectin, a glycoprotein, tripled in its contribution to the corona upon PS carboxylation.

This variation in protein composition of the NMs coronas could have significant consequences for biological fate and environmental impact. The three different NMs represent those which are found ubiquitously in 21st century life: silica is a common food additive, polystyrene makes up a significant portion of microplastics contaminating the environment, and titania is another common environmental contaminant coming from a wide range of sources such as sun cream and food colorants. The variations seen in these protein coronas suggest that when these NMs are exposed to humans, or released into the environment, intentionally or not, they each acquire their own unique protein fingerprint, each contributing to a unique array of assimilated characteristics. These new characteristics have the potential to induce toxicity in a range of organisms which may not usually display toxic response to compositionally equivalent bulk materials or to naïve NMs; however, the interactions between corona and organisms may initiate uptake, and thus subsequent associated toxicity. Alternatively, these acquired properties may attenuate or mitigate known toxic responses to NMs by reducing surface reactivity and thus preventing membrane damage and driving NM uptake via controlled, receptor-mediated processes. As such, there is a clear requirement to pursue the characterization of protein coronas across a range of biological and environmental matrices and NM compositions in order to comprehensively understand not only their clinical implications, but also their consequences for the environment. The use of highly sensitive, reproducible and versatile methods such as the one developed here utilizing CESI-MS will allow high-quality data to be generated to inform policies and aid in the development of ever more accurate and complex models simulating and predicting bio-nano interactions.

## 4. Conclusions

The work presented herein demonstrates the sequential development and validation of methods for isolating the NM protein corona. The finalized on-particle digestion approach offers the most extensive coverage of the protein corona in terms of the number of proteins and peptides identified and is also highly reproducible across an array of NM compositions and surface characteristics. This work also highlights the importance of including analytical and experimental replicates to enable an informed analysis of the reproducibility and quality of the data that is obtained for coronal characterization. Furthermore, the work introduces CESI-MS as a higher-throughput and very robust alternative to the more conventional LC-MS, thus further expanding on the current inventory of analytical platforms for NM corona characterization. 

Due to the extensive validation of the three most commonly utilized protein corona isolation techniques (i.e., methodologies for removing the NM-bound proteins from the particle surface for subsequent identification and quantification), it has become clear that some proteins are preferentially identified with each methodology. This additional insight into the impact of the selected corona isolation method on the corona identified enables researchers to understand their findings more thoroughly by accounting for methodological biases prior to analysis, or even once the data has been acquired. This new information may also explain why there are confounding reports in the literature as to whether protein corona correlates with NM cellular association and uptake or not [15,16,17], as depending on the method used to isolate the corona, a different sub-set of the total bound corona will have been reported, which may or may not contain proteins responsible for cellular attachment and internalization (e.g., albumin, transferrin, and many others [43,48]). Careful selection and detailed reporting of the corona isolation approach will enable the development of more accurate and precise models of protein corona formation and correlation of corona composition with cellular uptake and cellular impacts to be developed supporting the transition to a truly predictive understanding of the coronas roles in nanomaterials interactions, fate and impacts.

## Figures and Tables

**Table 1:** Proteins differing significantly (*p* < 0.05) in their abundance (following on-bead digestion from SiO_2_ NMs) depending upon the solubilizing agent used. Proteins written in bold showed a reduced abundance in the Rapigest SF™ approach.

Accession	Description	Coverage [%]	# Unique Peptides	Prot. Abund. Rapigest	Rapigest / none		Rapigest / Urea		Rapigest / both	
					Ratio	T test	Ratio	T test	Ratio	T test
P60709	Actin, cytoplasmic 1	57	7	8.13 × 10^8^	14.61	2.14 × 10^−4^				
P03950	Angiogenin	18	2	7.50 × 10^6^	2.62	6.21 × 10^−5^				
P01019	Angiotensinogen	16	6	1.03 × 10^7^			3.60	7.17 × 10^−3^		
**P02647**	**Apolipoprotein A-I**	93	69	1.01 × 10^10^			**0.25**	1.52 × 10^−2^		
**P02652**	**Apolipoprotein A-II**	74	15	3.32 × 10^8^			**0.05**	1.73 × 10^−2^		
**P02654**	**Apolipoprotein C-I**	51	10	1.98 × 10^7^	**0.03**	7.64 × 10^−4^	**0.07**	3.32 × 10^−3^		
**P02656**	**Apolipoprotein C-III**	63	9	8.25 × 10^7^			**0.10**	1.54 × 10^−2^		
**P02649**	**Apolipoprotein E**	82	40	3.10 × 10^8^			**0.36**	1.23 × 10^−2^		
**P08519**	**Apolipoprotein(a)**	7	2	5.99 × 10^6^	**0.20**	2.78 × 10^−3^				
**P00748**	**Coagulation factor XII**	12	9	9.71 × 10^7^	**0.24**	2.25 × 10^−3^	**0.47**	2.28 × 10^−3^	**0.49**	3.62 × 10^−5^
**P0C0L4**	**Complement C4-A**	54	3	3.13 × 10^7^			**0.43**	1.41 × 10^−4^		
**P00746**	**Complement factor D**	34	6	3.55 × 10^7^	**0.45**	1.58 × 10^−3^				
Q03591	Complement factor H-related protein 1	20	1	6.77 × 10^7^			3.00	3.51 × 10^−6^	3.70	1.11 × 10^−8^
Q86UX7	Fermitin family homolog 3	23	12	3.66 × 10^7^	5.82	4.40 × 10^−3^				
**Q15485**	**Ficolin-2**	4	1	1.29 × 10^6^			**0.50**	7.03 × 10^−52^		
A0A0A0MS15	Immunoglobulin heavy variable 3-49	17	2	6.29 × 10^6^					6.73	2.71 × 10^−27^
A0A0C4DH25	Immunoglobulin kappa variable 3D-20	22	1	3.59 × 10^6^			2.23	2.42 × 10^−3^		
**P24592**	**Insulin-like growth factor-binding prot. 6**	6	1	3.42 × 10^6^	**0.36**	1.12 × 10^−3^				
P05106	Integrin beta-3	10	6	2.45 × 10^7^			4.60	5.84 × 10^−3^		
P35579	Myosin-9	30	50	8.66 × 10^7^	10.57	8.20 × 10^−4^				
**P02760**	**Protein AMBP**	32	11	1.03 × 10^8^			**0.44**	8.91 × 10^−4^		
P14618	Pyruvate kinase PKM	18	6	7.20 × 10^6^	15.66	8.56 × 10^−15^				
**A0A096LPE2**	**SAA2-SAA4 readthrough**	50	11	3.59 × 10^8^	**0.44**	1.76 × 10^−3^	**0.48**	4.91 × 10^−5^		
Q9Y490	Talin-1	20	33	7.78 × 10^7^	4.77	3.69 × 10^−4^				
P07996	Thrombospondin-1	17	16	7.15 × 10^7^	13.02	2.78 × 10^−3^				
**P37802**	**Transgelin-2**	13	2	2.99 × 10^5^			**0.13**	1.75 × 10^−2^		
**Q9BYE2**	**Transmembrane protease serine 13**	1	1	1.56 × 10^7^	**0.18**	9.79 × 10^−5^	**0.30**	1.31 × 10^−4^		
**P02766**	**Transthyretin**	50	7	2.22 × 10^7^	**0.46**	1.73 × 10^−3^				

**Table 2:** Recovery of proteins that constitute the NM protein corona—both the fraction in solution and the fraction remaining on the NM post extraction.

	Experimental Component	TiO_2_	TiO_2_-PVP	TiO_2_-Dispex	PS	PS-COOH
On-particle digest	Summed MS signal intensity	1.0 × 10^11^	1.1 × 10^10^	7.7 × 10^10^	8.9 × 10^9^	3.6 × 10^10^
	High confidence proteins	80	81	83	77	86
Remaining in-gel	Summed MS signal intensity	5.1 × 10^7^	3.3 × 10^6^	1.2 × 10^8^	1.4 × 10^7^	2.6 × 10^8^
	% summed MS signal remaining	0.05%	0.03%	0.16%	0.15%	0.73%
	High confidence proteins	0	0	0	2	3
	Median remaining on particle *	0.05%	0.10%	0.09%	0.15%	0.80%

* calculated as the median of individual protein abundance ratios using peaks of the top 3 peptide intensities (Remaining on-particle/On-particle digestion).

**Table 3:** Protein abundance of total protein content of the corona across all 6 NMs investigated.

	PS-Carb		PS		TI		TI-PVP		TI-Dispex		Silica	
Protein	ppm	stdev	ppm	stdev	ppm	stdev	ppm	stdev	ppm	stdev	ppm	stdev
Fibrinogens	**13%**	0.2%	**6%**	1.2%	**43%**	1.1%	**41%**	0.8%	**9%**	0.6%	**17%**	2.9%
Apolipoproteins	**5%**	1.1%	**3%**	0.5%	**0%**	0.0%	**1%**	0.1%	**0%**	0.0%	**17%**	5.7%
Complement components	**7%**	1.5%	**1%**	0.6%	**8%**	0.7%	**6%**	0.3%	**8%**	0.3%	**12%**	1.9%
Immunoglobulin	**1%**	0.2%	**2%**	0.6%	**22%**	2.5%	**20%**	2.8%	**7%**	1.3%	**12%**	0.2%
Serum albumin	**15%**	0.6%	**61%**	1.7%	**1%**	0.0%	**0%**	0.0%	**1%**	0.1%	**8%**	1.4%
Vitronectin	**32%**	1.2%	**10%**	0.4%	**5%**	0.4%	**8%**	1.2%	**14%**	0.4%	**2%**	0.3%
Clusterin	**1%**	0.2%	**10%**	0.5%	**0%**	0.0%	**0%**	0.0%	**0%**	0.0%	**1%**	0.0%
Inter-alpha-trypsin inhibitor heavy chains	**3%**	0.3%	**1%**	0.1%	**1%**	0.5%	**1%**	0.0%	**0%**	0.1%	**4%**	0.5%
Kininogen-1	**3%**	0.0%	**0%**	0.0%	**1%**	0.1%	**3%**	0.6%	**2%**	0.4%	**3%**	0.3%
Histidine-rich glycoprotein	**7%**	2.5%	**0%**	0.0%	**1%**	0.2%	**1%**	0.1%	**0%**	0.0%	**1%**	0.1%
Alpha-2-HS-glycoprotein	**0%**	0.1%	**0%**	0.1%	**2%**	0.6%	**4%**	0.7%	**22%**	2.0%	**1%**	0.3%
Prothrombin	**0%**	0.2%	**0%**	0.0%	**5%**	0.4%	**7%**	0.6%	**21%**	0.3%	**0%**	0.0%
Serotransferrin	**0%**	0.0%	**0%**	0.1%	**0%**	0.0%	**0%**	0.0%	**0%**	0.0%	**4%**	1.7%
Plasminogen	**0%**	0.1%	**0%**	0.1%	**2%**	0.0%	**2%**	0.2%	**1%**	0.1%	**2%**	0.5%
Gelsolin	**0%**	0.2%	**0%**	0.0%	**1%**	0.3%	**2%**	0.1%	**1%**	0.0%	**1%**	0.2%
Beta-2-glycoprotein 1	**1%**	0.3%	**0%**	0.1%	**1%**	0.0%	**0%**	0.0%	**0%**	0.1%	**1%**	0.0%
Vitamin D-binding protein	**0%**	0.1%	**2%**	1.6%	**0%**	0.0%	**0%**	0.0%	**0%**	0.0%	**0%**	0.0%
Vitamin K-dependent protein S	**0%**	0.0%	**0%**	0.0%	**1%**	0.1%	**1%**	0.1%	**1%**	0.1%	**0%**	0.0%
Plasma kallikrein	**1%**	0.1%	**0%**	0.0%	**1%**	0.1%	**1%**	0.1%	**1%**	0.2%	**0%**	0.0%
Hemopexin	**0%**	0.0%	**0%**	0.0%	**0%**	0.0%	**0%**	0.0%	**0%**	0.0%	**2%**	0.2%
others	**9%**	0.3%	**3%**	0.5%	**6%**	1.0%	**5%**	0.0%	**9%**	0.5%	**14%**	1.3%
												
	Colour code:		**0%**	5.0%	**10%**	30.0%	**60%**					

## Supplementary Materials

The following are available online at https://www.mdpi.com/2079-4991/9/6/898/s1, Supplementary materials, SI-1: DLS and zeta potential data, SI-2: Initial on-particle digest metadata, SI-3: CESI-MS/MS reproducibility metadata, SI-4: CESI-MS/MS vs nLC-MS/MS comparison, SI-5: CESI-MS/MS vs nLC-MS/MS metadata, SI-6: Optimisation of enzymatic on-particle digest metadata, SI-7: Pairwise comparison of protein abundances in SiO_2_ NM corona metadata, SI-8: Protein corona isolation efficiency metadata, SI-9: On-particle digest vs in-gel and in-solution digest metadata, SI-10: Protein corona composition on-particle vs in-gel digest metadata, SI-11: Characterisation of protein corona recovery across a range of particles and surface modifications metadata.
