# Supplementary material for: Corona Isolation Method Matters: Capillary Electrophoresis Mass Spectrometry Based Comparison of Protein Corona Compositions Following On-Particle versus In-Solution or In-Gel Digestion
Source: Nanomaterials (Basel). 2019 Jun 20;9(6):898. doi: 10.3390/nano9060898 (PMC6631359; doi:10.3390/nano9060898)
Supplement: Supplementary file 1 [file nanomaterials-09-00898-s001.zip › Supplementary Materials/Supplementary Materials SI-4.docx]

**Corona isolation method matters: capillary electrophoresis mass spectrometry based comparison of corona compositions following on-particle versus in-solution or in-gel digestion**

Klaus Faserl^1#^, Andrew J. Chetwynd^2#^, Iseult Lynch^3^, James A. Thorn^2^, Herbert H. Lindner^1^*

^1^Division of Clinical Biochemistry, Medical University of Innsbruck, Innrain 80-82, A-6020, Innsbruck, Austria

^2^ AB Sciex UK Ltd., Phoenix House, Lakeside Drive, Warrington, Cheshire, WA1 1RX, UK

^3^ School of Geography Earth and Environmental Sciences, University of Birmingham, Edgbaston, Birmingham, B15 2TT, UK

Supplementary Materials SI-4

Contents

Page 2) NanoLC-MS/MS methods

Page 3) Comparison of CE-MS/MS to LC-MS/MS

**NanoLC-MS/MS methods**

Nano-LC-MS peptide analyses were performed on a Dionex UltiMate 3000 nano-HPLC system (Thermo Scientific, Bremen, Germany) coupled via Nanospray Flex ion source to a Thermo Scientific Q Exactive HF mass spectrometer. Samples were injected as 18 μL aqueous solutions containing 0.1% formic acid. Peptides were concentrated on a PepMap100 C18 trap column (300 µm i.d., 5 mm length, 5μm particles with 100 Å pore size) and separated on a fritless pulled fused-silica capillary column (100 µm i.d., 20 cm length) packed with 2.4 µm reversed-phase material (ReproSil-Pur C18-AQ with 120 Å pores). The gradient (solvent A: 0.1% formic acid; solvent B: 0.1% formic acid in 85% acetonitrile) was initially isocratic at 4% B for 4 min. Solvent B was then increased linearly to 30% within 113 min, and to 100% during 5 min, was kept at 100% for 5 min, and reduced linearly to 4% within 5 min. A flowrate of 300 nL/min was applied.

After peptide separation the HPLC system was washed by injecting 20 µl of 10% trifluoroethanol in acetonitrile and performing an isocratic separation for 5 min using a solvent B concentration of 4%. This procedure was performed twice. Sample carry over was controlled by injecting 5 µl of a water blank and performing a shorter separation using a gradient that starts at 4% solvent B, which was increased to 50% during 40min and to 100% within 5min.

Settings for the Q-Exactive HF mass spectrometer were as described for the CESI-MS analyses, except that the twenty highest precursors were selected for MS/MS fragmentation.

**Comparison of nanoLC-MS/MS to CESI-MS/MS**

nLC-MS/MS is the workhorse of proteomics and indeed is the most common mass spectrometric method used in protein corona characterisation.[1] However in recent years in proteomics CESI-MS/MS has grown in popularity as an orthogonal separation technique better suited the highly polar peptides which are lost in the solvent front of traditional LC runs, or for very large peptides which do not elute from an LC column.[2] While a number of comparisons have been performed in the proteomics literature[3,4] this is the first for protein corona characterisation and offers additional information about the CESI-MS/MS technique this new research area.

Initially the migration/retention time reproducibility was assessed for both platforms, here CESI-MS/MS outperformed the LC-MS/MS with a mean RSD of 0.3% vs 0.59% for the LC-MS/MS both of these values represent very high degrees of reproducibility though the CESI migration time stability is nearly twice that of the nLC-MS/MS. In addition, the peak area reproducibility for peptides was also assessed for both platforms with average RSDs of 16.45% for LC-MS/MS and 16.28% for CESI-MS/MS which represents good repeatability of the analysis for both platforms.

When it came to the number of proteins and peptides identified the LC-MS/MS was able to identify 325 proteins and 3606 peptides compared to 184 and 2031 respectively for CESI-MS/MS (Figure 1). This result is congruent with the literature and is predominantly a result of the greater loading capacity of LC-MS/MS, in this comparison 21.74 times more sample was injected compared to the CE-MS/MS. In addition, a fritless nanoLC emitter was utilized which allows for a very sensitive analysis. However, most protein corona papers detail only the top 10 or 20 most abundant proteins, few studies report as many proteins identified as this CESI-MS/MS approach has despite using LC-MS/MS. This is possibly a result of poor attention to detail with sample preparation.


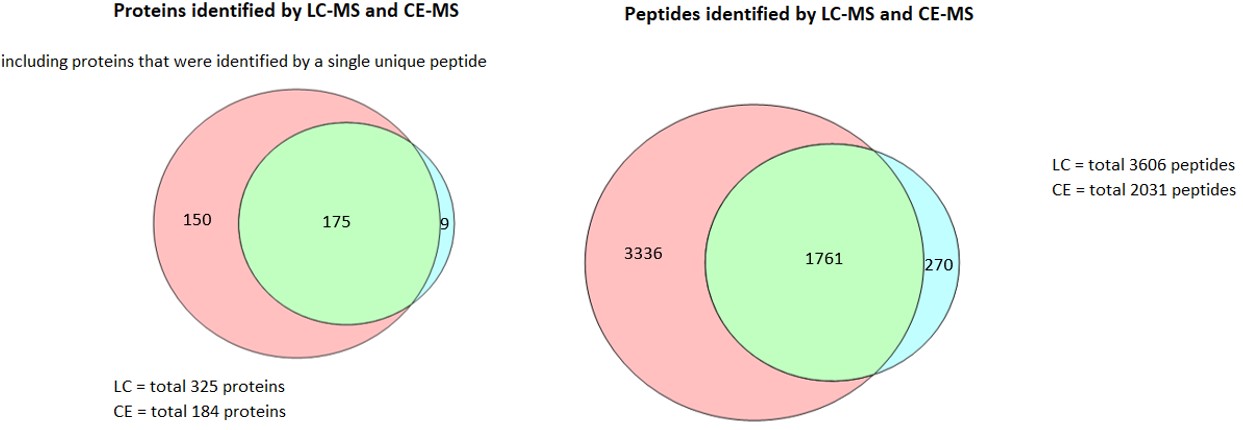


Figure 1: Numbers of proteins and peptides detected on the LC-MS/MS platform vs the CESI-MS/MS

The most significant difference between the CESI-MS/MS and the LC-MS/MS is the degree of carry over between samples. As seen in the main manuscript in Figure 3 the CESI-MS/MS shows no evidence of any carry over, with a electropherogram devoid of peaks and no peptides being detected in a solvent blank injection immediately following the sample injection. However, with LC-MS/MS carry over is a serious consideration as peptides physically interact with the C18 stationary phase of LC columns, whereas in CESI-MS/MS there are little to no interactions between peptides and the capillary particularly when neutral capillaries are used. In Figure 2, it is clear that a third of the initial peptides from the sample can be detected and identified in a solvent blank injection. A trend that is repeat with a second solvent blank in which a third of the peptides remaining from the first blank can still be identified. A similar trend can also be observed in the summed peptide signal intensity in Figure 3. The presence of carryover can complicate data analysis and lead to erroneous conclusions wither with peptide identifications (identified as present instead of absent) or quantification. As a result, significant system clean up steps are taken with LC-MS/MS as described in the methods compared to the CESI-MS/MS which is capable of running back to back samples. As such there is a significant impact on same throughput, CESI-MS/MS allows a sample injection approximately every 50 minutes, with the LC-MS/MS this is reduced to 1 sample every 187 minutes. Thus, reducing the potential daily throughput to be decreased from 28 samples/day with CESI-MS/MS to just 7 with LC-MS/MS. Given that it is typical that only the top 20 most intense proteins are reported in corona studies as opposed to a more comprehensive quantification and discussion of all proteins identified adopting a CESI-MS/MS approach would significantly increase sample through put for the same mass spectrometer time thus greatly reducing sample costs while maintaining the same high degree of reproducibility of the LC-MS/MS analysis.

Figure 2: The number of peptides identified in the initial sample and subsequent two blank solvent injections

Figure 3: Logged peak intensities for initial sample injection and subsequent solvent blanks

**References**

1. Fischer, R.; Bowness, P.; Kessler, B.M. Two birds with one stone: Doing metabolomics with your proteomics kit. *Proteomics* **2013**, *13*, 3371–3386.

2. Chetwynd, A.J.; Guggenheim, E.J.; Briffa, S.M.; Thorn, J.A.; Lynch, I.; Valsami-Jones, E. Current application of capillary electrophoresis in nanomaterial characterisation and its potential to characterise the protein and small molecule corona. *Nanomaterials* **2018**, *8*, 99.

3. Faserl, K.; Sarg, B.; Kremser, L.; Lindner, H. Optimization and evaluation of a sheathless capillary electrophoresis-electrospray ionization mass spectrometry platform for peptide analysis: comparison to liquid chromatography-electrospray ionization mass spectrometry. *Anal. Chem.* **2011**, *83*, 7297–7305.

4. Sarg, B.; Faserl, K.; Kremser, L.; Halfinger, B.; Sebastiano, R.; Lindner, H.H. Comparing and Combining Capillary Electrophoresis Electrospray Ionization Mass Spectrometry and Nano–Liquid Chromatography Electrospray Ionization Mass Spectrometry for the Characterization of Post-translationally Modified Histones. *Mol. Cell. Proteomics* **2013**, *12*, 2640–2656.
